# Inhibition of β-catenin and STAT3 with a curcumin analog suppresses gastric carcinogenesis in vivo

**DOI:** 10.1007/s10120-014-0434-3

**Published:** 2014-10-18

**Authors:** Yoshihiko Uehara, Masahiro Inoue, Koji Fukuda, Hiroyuki Yamakoshi, Yoshio Hosoi, Hiroaki Kanda, Masanobu Oshima, Yoshiharu Iwabuchi, Hiroyuki Shibata

**Affiliations:** 1 Department of Cell Biology, Graduate School of Medicine, Tohoku University, Sendai, Japan; 2 Department of Clinical Oncology, Graduate School of Medicine, Akita University, Hondo 1-1-1, Akita, 010-8543 Japan; 3 Department of Organic Chemistry, Graduate School of Pharmaceutical Science, Tohoku University, Sendai, Japan; 4 Division of Pathology, Cancer Institute, Japanese Foundation for Cancer Research, Tokyo, Japan; 5 Division of Genetics, Cancer Research Institute, Kanazawa University, Kanazawa, Japan

**Keywords:** Analog, β-Catenin, Curcumin, Gastric cancer, Signal transducer and activator of transcription 3

## Abstract

**Background:**

Potent chemotherapy for advanced gastric cancer has not been completely established. Many molecularly targeted therapies are under investigation, but their therapeutic outcomes are not promising because they do not target specific and/or critical targets of gastric carcinogenesis. Although the molecular basis of gastric carcinogenesis remains poorly understood, nuclear localization of β-catenin was observed in approximately 50 % of gastric cancer specimens. Recent studies have suggested that activation of signal transducer and activator of transcription 3 (STAT3) contributes to gastric carcinogenesis in a mouse model. A newly synthesized curcumin analog has inhibitory potential against β-catenin and STAT3.

**Methods:**

Using a transgenic mouse model of gastric cancer in which β-catenin, cyclooxygenase 2, and microsomal prostaglandin E synthase 1 activation is induced, we examined a curcumin analog with the most enhanced potential for treating gastric cancer through oral administration. Inhibition of these targets was demonstrated using microarray and immunohistochemical analyses.

**Results:**

The curcumin analog GO-Y031 decreased the incidence of gastric carcinogenesis to 54.5 % of that of the control (50.0 % vs 91.7 %, *p* = 0.043), and tumor size was reduced to 51.6 % of that of the control (1.6 mm vs 3.1 mm, *p* = 0.03). β-Catenin and STAT3 levels were suppressed to 26.2 % (*p* = 0.00023) and 44.8 % (*p* = 0.025), respectively, of those of the control. Moreover, macrophage infiltration was suppressed with GO-Y031.

**Conclusion:**

β-Catenin and STAT3 can be pharmacologically inhibited in vivo with a curcumin analog, which effectively inhibits β-catenin and STAT3.

**Electronic supplementary material:**

The online version of this article (doi:10.1007/s10120-014-0434-3) contains supplementary material, which is available to authorized users.

## Introduction

Gastric cancer is a malignancy with a high incidence, particularly in Asia [1, 2]. In three Asian countries—Japan, Korea, and China—gastric cancer accounts for approximately 60 % of new cancer patients [1, 2]. However, the prevention and early detection of gastric cancer remains difficult, and surgery is the only current treatment for this cancer. Advanced-stage gastric cancer is diagnosed in many patients, and recurrence after surgery is frequent. Systemic chemotherapy is only the method for treating such patients. No effective agents have been developed for treating gastric cancer to date, except for capecitabine, cisplatin, and trastuzumab combination therapy [3]. However, even with this combination therapy, the median overall survival time was only 13.8 months for human epidermal growth factor receptor 2 (HER2)-positive patients, which is lesser than that for patients with colorectal cancer [3]. Because HER2-positive gastric cancer accounts for only 7–34 % of gastric cancer cases [4], agents with other molecular targets are being eagerly investigated. These include ramucirumab [5] and apatinib [6], which target vascular endothelial growth factor receptor (VEGFR) 2, and sorafenib [7], which acts as a multi-tyrosine kinase inhibitor of VEGFR2 and VEGFR3, platelet-derived growth factor receptor, and RAF kinase. However, these targets are not specifically activated in gastric carcinogenesis.

The unsatisfactory treatment of gastric cancer reflects insufficient understanding of the associated molecular carcinogenesis. In 1994, *Helicobacter pylori* infection was classified as a definite carcinogen by the International Agency for Research on Cancer [8]. Hence, the mechanisms by which chronic infectious stimuli contribute to this malignancy are under investigation. Recently, it was shown that the CagA protein of *H. pylori* facilitates nuclear localization of β-catenin in gastric cancers [9]. β-Catenin is a component of Wnt signaling, and its nuclear localization has been observed in 30–58 % of clinical gastric carcinoma specimens [10, 11]. Furthermore, the activation of Wnt signaling by inflammatory stimuli may induce gastric carcinomas in the K19-Wnt1/C2mE transgenic mouse model of gastric cancer (Gan mouse), which expresses transgenic Wnt1, cyclooxygenase (COX) 2, and microsomal prostaglandin E synthase-1 under the control of the K19 promoter [12]. Thus, β-catenin may play a significant role in gastric carcinogenesis.

Hyperactivation of signal transducer and activator of transcription 3 (STAT3) has been observed in many types of cancers [13]. Moreover, in a mouse model of gastric cancer, STAT3 activation promoted carcinogenesis via Toll-like receptor 2 without inflammatory signaling [14]. STAT3 may also participate in gastric carcinogenesis following activation by COX2 in many cancers, such as non-small-cell carcinoma, cholangiocarcinoma, and glioblastoma [15–17].

In this study, we synthesized novel curcumin analogs and showed that they are potent inhibitors of β-catenin in vitro and in vivo [18, 19]. In our previous studies, phosphorylation of STAT3 was decreased following treatment of pancreatic cancer, breast cancer [20], and multiple myeloma cell lines [21] with the novel curcumin analogs (1E,4E)-1,5-bis-(3,5-bismethoxymethoxyphenyl)penta-1,4-dien-3-one (GO-Y030) and (1E,4E)-1-(4-hydroxy-3,5-dimethoxyphenyl)-5-(3,4,5-trimethoxyphenyl)-penta-1,4-dien-3-one (GO-Y078). Moreover, GO-Y030 suppressed the growth of colorectal cancer stem cells by inhibiting phosphorylated STAT3 (pSTAT3) [22], suggesting that curcumin analogs have anti-inflammatory activities such as those demonstrated with curcumin [23, 24].

Among 86 newly synthesized curcumin analogs, GO-Y030, (1E,4E)-1,5-bis(3,5-dimethoxy-4-methoxymethoxyphenyl)pentadien-3-one (GO-Y031), and GO-Y078 had the most potent antitumor activities [25]. Moreover, in the *Apc*580D/+ mouse model of colorectal tumorigenesis, GO-Y030 and GO-Y031 suppressed adenoma formation by inhibiting β-catenin [19]. However, no studies have shown whether curcumin analogs have antitumor activity under the low-pH conditions in the stomach. Thus, we investigated the effects of orally administered GO-Y031 in the Gan mouse model of gastric cancer, and we demonstrated effective blockade of β-catenin and promising anticancer efficacy. The present data show that the inhibition of β-catenin and/or STAT3 suppresses gastric carcinogenesis, suggesting that these molecules are efficacious targets for the medicinal treatment of gastric cancer.

## Materials and methods

### Chemicals

**Fig. 1:**
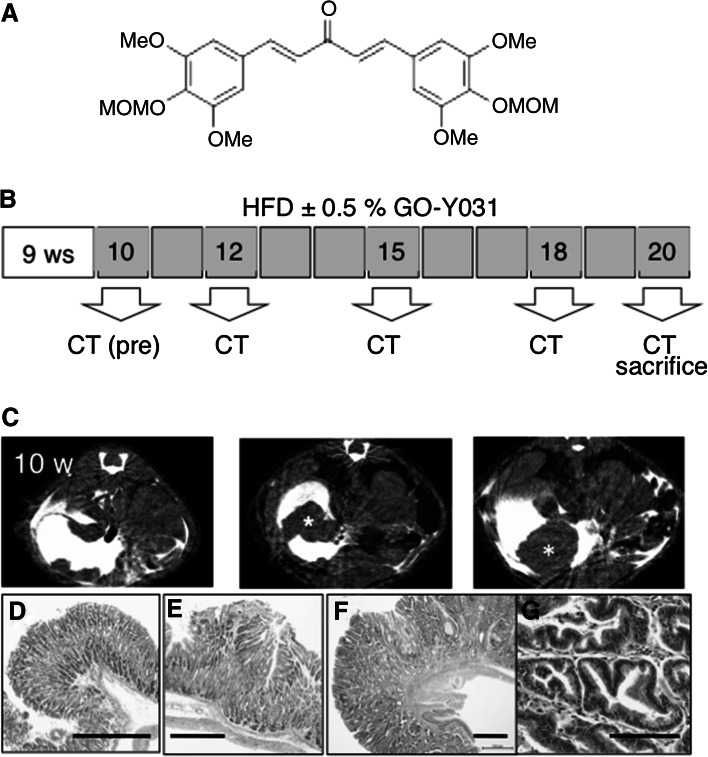
Experimental overview. **a** Chemical structure of GO-Y031. *MeO* indicates a methoxy group and *MOMO* indicates a methoxymethoxy group. **b** The experimental schedule. Computed tomography (*CT*) gastrograms were conducted at 10, 12, 15, 18, and 20 weeks of age. **c** CT images of the stomachs of Gan mice; **d**–**g** Histopathology micrographs corresponding to CT images: **d**, **c** *left*; **e**, **c** *middle*; **f** and **g**, **c** *right*. *Bars* represent 500 µm in **d**–**f** and 100 µm in **g**. An *asterisk* indicates a gastric tumor. *HFD* high-fat diet, *w* weeks, *ws* weeks

The synthesis of GO-Y031 has been described previously (Fig. 1a) [18]. The high-fat diet (HFD) HFD32 was purchased from CLEA Japan (Tokyo, Japan).

### Mouse model

K19-Wnt1/C2mE Gan mice were obtained by crossing K19-Wnt1 and K19-C2 mE mice. Genotyping was confirmed as described previously [11]. Daily, Gan mice were fed 5 g of HFD alone or HFD plus 0.5 % (w/w) GO-Y031 from 10 weeks of age (Fig. 1b) and were killed and examined at 20 weeks of age. All animal experiments were performed humanely and complied with the guidelines set by Tohoku University and were approved by the associated ethics committee.

### X-ray computed tomography gastrograms

X-ray computed tomography (CT) images of gastric tumors in live mice were examined using a Latheta LCT-200 instrument (Hitachi Aloka, Tokyo, Japan) at 10–12 (pretreatment), 13–15, 16–18, and 20 weeks of age. The radiopaque contrast agent Iopamiron 300 (Bayer Pharma, Osaka, Japan) was administered to mice under anesthesia via gavage and intraperitoneal injection (0.2 ml each, diluted 1:5) immediately before CT scanning.

### Immunohistochemistry

After the mice had been killed, mouse stomachs were resected and fixed in 10 % neutral buffered formalin, and were then dipped into series of ethanol solutions (70–100 %) and embedded in paraffin. Immunohistochemistry (IHC) was conducted as described previously [19] using the following antibodies: anti-mouse β-catenin (1:1500, C2206, rabbit antiserum, Sigma-Aldrich, Tokyo, Japan), anti-mouse Ki-67 (2 μg/ml, ab15580, rabbit polyclonal antibody, Abcam, Tokyo, Japan), anti-mouse STAT3 (1:500, CST Stat3; 124H6, mouse monoclonal antibody; Cell Signaling Technology Japan, Tokyo, Japan), anti-mouse pSTAT3 (1:100, Tyr705; D3A7, XP rabbit monoclonal antibody, Cell Signaling Technology Japan), anti-mouse p53 protein (1:2,000, CM5, rabbit polyclonal antibody, Vector Laboratories, Burlingame, CA, USA), anti-mouse c-Myc (1:200, 9E10; sc-40, mouse monoclonal antibody, Santa Cruz Biotechnology, Dallas, TX, USA), and anti-mouse δ_1_-catenin [1:200, EPR357(2), ab92514, rabbit monoclonal antibody, Abcam]. IHC for β-catenin and Ki-67 was conducted by Genostaff (Tokyo, Japan). IHC for CD44 and F4/80 (MCA497R clone A3-1, Serotec, Oxford, UK) was conducted as described previously [11, 26].

### Microarray analysis

In the present mouse model, tumors have a protruding appearance and are easily distinguished from the surrounding nontumorous and normal membranes. Thus, normal mucosa was collected during stereomicroscopic examinations.

Total RNA was extracted from background epithelia of three independent mouse stomachs. Equal amounts of RNA from each treatment group were mixed and analyzed. Expression analysis was conducted using the CodeLink™ mouse whole genome bioarray (Applied Microarrays, Tempe, AZ, USA), and data analysis was outsourced to Filgen^®^ (Nagoya, Japan). Data were analyzed using Microarray Data Analysis Tool version 3.2 (Filgen^®^).

### Statistical analysis

Data are shown as the mean ± standard deviation. Differences between groups were identified using Fisher’s exact probability test and Student’s *t*-test with StatMate III version 3.14 (ATMS, Tokyo, Japan).

## Results

### Antitumor activity of the curcumin analog GO-Y031 in a mouse model of gastric cancer

**Fig. 2:**
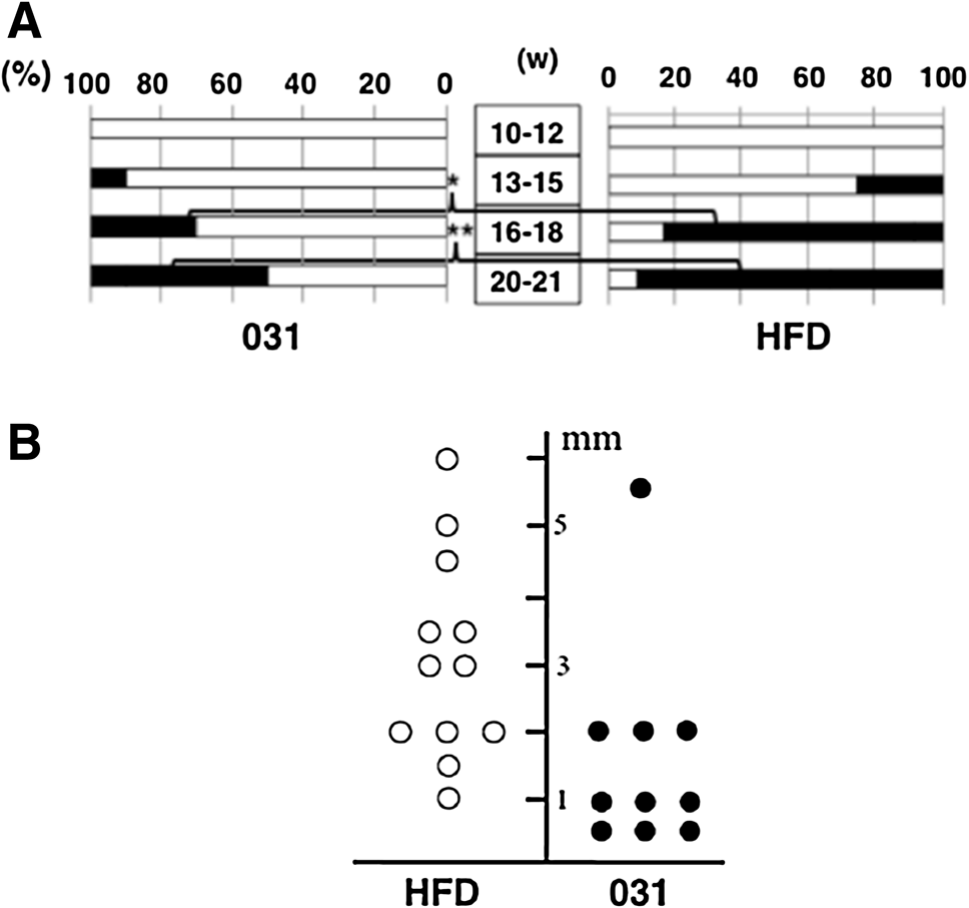
GO-Y031-mediated tumor suppression. **a** Chronological incidence of gastric tumors in HFD-fed Gan mice also given GO-Y031. Tumor incidence is shown as *bars*. *Asterisks* indicate significant differences. **b** Actual heights of stomach tumors in Gan mice at 20 weeks of age. *w* weeks

Because GO-Y031 is hydrophobic, HFD32 was used to prepare homogeneous mixtures of pellets as described previously [19]. Tumor incidence in HFD-fed Gan mice was examined using CT gastrograms (Fig. 1c), and tumor formations were identified in live mice. All tumors were adenocarcinomas (Fig. 1d–g), and tumor formation was examined chronologically (Fig. 2a). No tumors were observed in mice aged 10–12 weeks (*n* = 12). However, at 13–15, 16–18, and 20 weeks of age, 25.0, 83.3, and 91.7 % of HFD-fed Gan mice, respectively, harbored gastric tumors. Tumor incidence in HFD-fed Gan mice was suppressed by supplementation with 0.5 % GO-Y031 (Fig. 2a), and at 16–18 and 20 weeks of age, only 30.0 % (*n* = 10, *p* = 0.017) and 50.0 % (*p* = 0.043) of mice, respectively, had gastric tumors. The average tumor height in HFD-fed Gan mice was 3.1 ± 1.5 mm, whereas that in HFD-fed Gan mice also given 0.5 % GO-Y031 was only 1.6 ± 1.5 mm (Fig. 2b, *p* = 0.03). These data indicate that GO-Y031 may significantly suppress both tumor incidence and growth in Gan mice. In subsequent experiments, we examined anti-inflammatory effects of GO-Y031 in terms of epithelial thickness. However, no differences were found between the two treatment groups, indicating that GO-Y031 exerts potent antitumor activity without acting as an anti-inflammatory agent (data not shown).

### Molecular targets of curcumin analogs

**Fig. 3:**
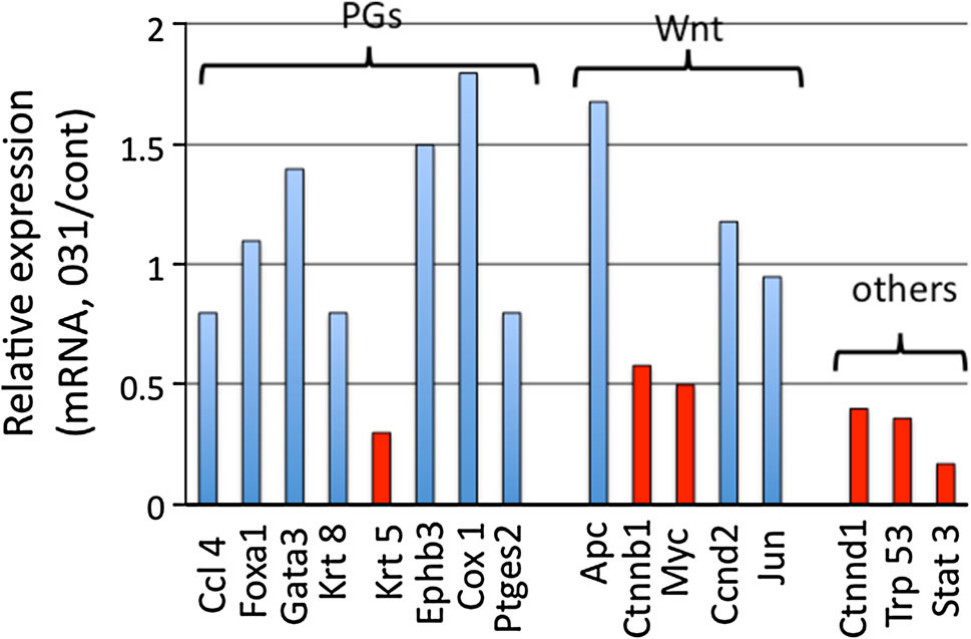
Comparison of expression profiles between normal mucosa of GO-Y031-treated Gan mice and control mice. Data are expressed relative to the control. *Red bars* indicate suppression to below 50 % of the level of the control. Transcripts related to prostaglandin and cytokine signaling are shown *below PGs* and transcripts related to Wnt signaling are shown *below Wnt*. *Apc* adenomatous polyposis coli, *Ccl 4* chemokine (C–C motif) ligand 4, *CCnd2* cyclin D2, *Cox 1* cyclooxygenase 1, *Ctnnb1* β-catenin, *Ctnnd1* δ_1_-catenin, *Ephb3* ephrin type B receptor 3, *Foxa1* forkhead box protein A1, *Gata3* GATA-binding protein 3, *Krt 5* keratin 5, *Krt 8* keratin 8, *mRNA* messenger RNA, *Ptges2* prostaglandin E synthase 2, *Stat 3* signal transducer and activator of transcription 3, *Trp 53* p53

Curcumin and its analogs have multiple potential targets [27]. Thus, we examined molecular targets of GO-Y031 using expression microarrays. Messenger RNA from background epithelia of 20-week-old HFD-fed Gan mice was analyzed in the presence and absence (control) of orally administered 0.5 % GO-Y031. Transcripts with signals below the noise level were excluded, and expression of the remaining transcripts was compared between treatment and control mice. Among 36,000 transcripts, 154 were upregulated to more than 200 % of the level of the control, whereas 351 were downregulated to less than 50 % of the level of the control in HFD-fed Gan mice also given 0.5 % GO-Y031. Transcripts that have been previously analyzed in Gan, K19-Wnt1, and K19-C2mE mice and those related to inflammation are shown in Fig. 3 [12, 28]. In addition, transcripts that were significantly regulated by treatment with GO-Y031 and were related to Wnt signaling or malignancy are indicated in Fig. 3. Treatment with GO-Y031 did not affect the expression of transcripts related to inflammation, including COX1, prostaglandin E synthase 2, keratin 8, chemokine (C–C motif) ligand 4, forkhead box protein A1, GATA-binding protein 3, and ephrin type B receptor 3 (Fig. 3). However, GO-Y031 treatment decreased the transcription of β-catenin, c-Myc, STAT3, and δ_1_-catenin to 58, 50, 17, and 40 % of the level of the control, respectively (Fig. 3). The expression of p53 was decreased to 36 % of the level of the control (Fig. 3).

### Suppression of β-catenin by the curcumin analog GO-Y031

**Fig. 4:**
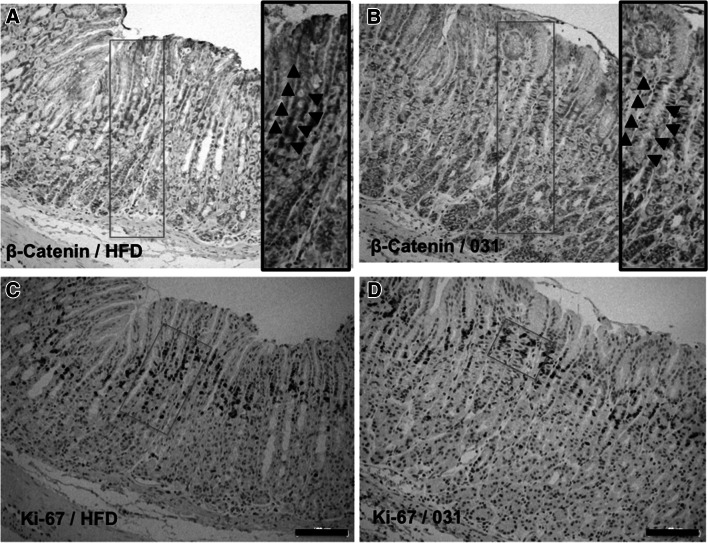
Immunohistochemical analyses of Wnt signaling. **a** Expression of β-catenin in normal mucosa from HFD-fed Gan mice. The *inset* shows a higher magnification of the *red rectangular area*, and *triangles* indicate cells that were positive for nuclear and cytosolic β-catenin. **b** β-Catenin in normal mucosa from GO-Y031-treated Gan mice. The *inset* shows a higher magnification, and *triangles* indicate cells with decreased cytosolic β-catenin staining. **c** Ki-67 expression in normal mucosa from HFD-fed Gan mice. The *red rectangle* shows the Ki-67-positive intracellular area. **d** Ki-67 expression in normal mucosa from GO-Y031-treated Gan mice. The *red rectangle* shows decreased Ki-67-positive areas. The *bars* represent 100 μm

**Fig. 5:**
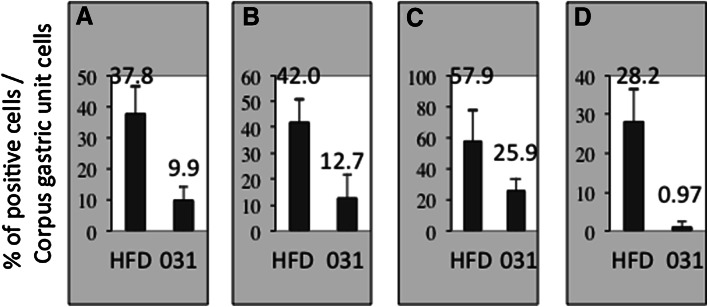
Quantitative analyses of immunoreactivity is presented as a percentage of positive cell areas. **a** Expression of β-catenin. **b** Expression of Ki-67. **c** Expression of STAT3. **d** Expression of phophorylated STAT3 (pSTAT3). The *bars* represent standard deviations

IHC indicated that cytosolic β-catenin-positive cells in the corpus gastric unit were less abundant in the background epithelia of Gan mice fed HFD plus 0.5 % GO-Y031 than in those of Gan mice fed HFD alone (9.90 ± 4.58 % vs 37.8 ± 8.77 %, *p* = 0.00023; Figs. 4a, b, 5a). Importantly, nuclear staining of β-catenin was observed in gastric cancer tissues (Fig. S1a). Because accumulated cytosolic β-catenin is transferred to the nucleus and acts as an oncogenic transactivator that promotes hyperproliferation, we examined Ki-67 expression as a marker of growth. Although 12.7 ± 9.38 % of cells in the corpus gastric unit in 0.5 % GO-Y031-treated HFD-fed Gan mice were Ki-67 positive, 42.0 ± 8.81 % of these cells in control mice were Ki-67 positive (*p* = 0.00094, Figs. 4c, d, 5b). Similarly, Ki-67 signals were abundantly observed in gastric cancer tissues (Fig. S1b). These data indicate that orally administered GO-Y031 acts as a β-catenin inhibitor in the stomach and may suppress the incidence and growth of malignant cells. Importantly, β-catenin levels were not reduced in nontransgenic wild-type mice (Fig. S2a).

### Suppression of STAT3 activation by the curcumin analog GO-Y031

**Fig. 6:**
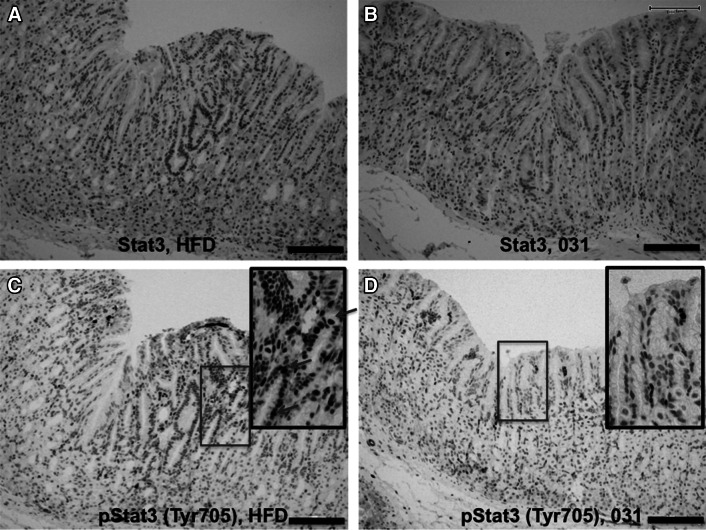
Immunohistochemical analyses of STAT3. **a** Expression of STAT3 in normal mucosa of HFD-fed Gan mice. **b** STAT3 in normal mucosa from GO-Y031-treated Gan mice. **c** Expression of pSTAT3 (Tyr705) in normal mucosa from HFD-fed Gan mice. The *inset* shows a higher magnification of the *red rectangular area*. *Red arrows* indicate typical nuclear pSTAT3 signals. **d** Expression of pSTAT3 (Tyr705) in normal mucosa from GO-Y031-treated Gan mice. The *inset* shows a higher magnification of the *red rectangular area*. No signals were detected in epithelial cells. The *bars* represent 100 μm

Expression analyses of STAT3 were confirmed by IHC. In these experiments, 25.9 ± 8.14 % of the cells in the corpus gastric unit were STAT3 positive in 0.5 % GO-Y031-treated HFD-fed Gan mice, whereas 57.8 ± 19.9 % of the cells were STAT3 positive in control mice (*p* = 0.025, Figs. 5c, 6a). Moreover, STAT3 signals were more pronounced in the cytosol and nucleus of cells from control mice than in the cytosol and nucleus of cells from 0.5 % GO-Y031-treated HFD-fed Gan mice. Nuclear transfer of STAT3 is dependent on the phosphorylation of STAT3 at tyrosine 705, which results in the activation of STAT3 [29]. Hence, IHC was conducted using a specific antibody for this pSTAT3. In these experiments, 0.97 ± 1.67 % of the cells in the corpus gastric units of 0.5 % GO-Y031-treated HFD-fed Gan mice were pSTAT3 positive, whereas 28.2 ± 8.36 % of the cells in control mice were pSTAT3 positive (*p* = 0.0052, Figs. 5d, 6c, d). STAT3 and pSTAT3 signals were abundantly observed in gastric cancer tissues (Fig. S1c, d). Hence, in agreement with the present microarray data, treatment with GO-Y031 significantly decreased the numbers of pSTAT3-activated cells in mice with gastric cancer, indicating that this curcumin analog may inhibit the phosphorylation of STAT3. Importantly, pSTAT3 signals were not detected in nontransgenic wild-type mice (Fig. S2c).

**Fig. 7:**
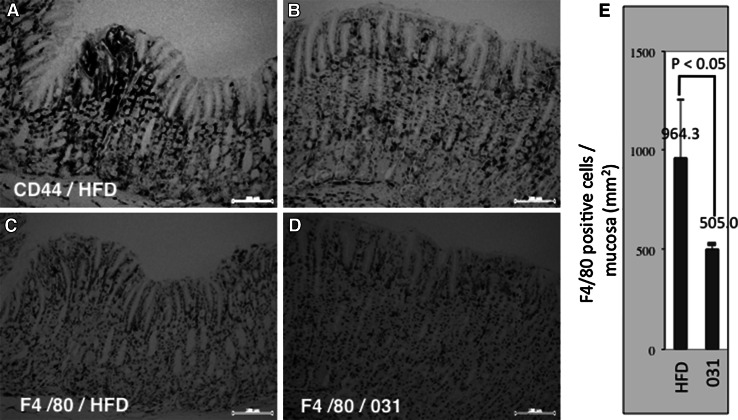
Immunohistochemical analyses of CD44 and F4/80. **a** Expression of CD44 in normal mucosa from HFD-fed Gan mice. **b** CD44 expression in normal mucosa from GO-Y031-treated Gan mice. **c** F4/80 expression in normal mucosa from HFD-fed Gan mice. **d** F4/80 expression in normal mucosa from GO-Y031-treated Gan mice. **e** Numbers of F4/80-positive macrophages in five randomly selected areas from each treatment group. The *bars* represent standard deviations

CD44 is a major cell adhesion molecule that participates in various physiological events, including lymphocyte homing, wound healing, and cell migration as well as in cancer cell growth and metastasis [30]. Accordingly, CD44 has been implicated in cancer stem cell maintenance during gastric carcinogenesis [26]. Recently, it was shown that CD44 is upregulated by β-catenin [9]. Thus, we examined the expression of CD44 in the background mucosa of Gan mice. Although CD44 messenger RNA was not detectable in the present microarray experiments, compared with control mice, areas with CD44-positive IHC signals were reduced in 0.5 % GO-Y031-treated HFD-fed Gan mice (Fig. 7a, b), indicating that GO-Y031 reduces CD44 expression in the background epithelia of Gan mice.

### Other candidate protein targets

IHC for p53 (Fig. S3), δ_1_-catenin, and c-Myc did not show any differences between treatment and control groups (data not shown), although p53 was undetectable in gastric cancer tissues from Gan mice (Fig. S1e). These observations were inconsistent with the present microarray data, which did not confirm the downregulation of these genes by GO-Y031. In further IHC experiments with an F4/80 antibody [11], macrophage infiltration was abundant in gastric mucosa of Gan mice, reflecting increased prostaglandin E_2_ expression [11]. Compared with control HFD-fed Gan mice, treatment of Gan mice with GO-Y031 significantly suppressed macrophage infiltration (Fig. 7c–e), indicating that GO-Y031 has anti-inflammatory effects that may be similar to those of curcumin.

### Safety issues

The safety of GO-Y031 was confirmed in the present study, with no agent-related adverse events, as shown previously [19].

## Discussion

Transgenic expression of β-catenin in gastric epithelia may induce gastric cancer, and in this study, we clearly showed that suppression of β-catenin inhibits gastric carcinogenesis. Oshima established the present mouse model, and cancers in these Gan mice were recently shown to belong to the intestinal type according to Laurén’s classification [31]. In this study, we confirmed that β-catenin is a candidate target for the treatment of gastric cancer and showed the therapeutic potential of the curcumin analog GO-Y031. Initially, suppression of STAT3 and β-catenin expression was observed in the Gan mouse model of gastric cancer, and subsequent immunohistochemical analyses indicated suppression of STAT3 phosphorylation. Previous reports showed that β-catenin influences STAT3 and vice versa [32–35]. In our previous studies, the curcumin analog GO-Y030 inhibited β-catenin in the mouse colonic epithelium, inhibited STAT3 phosphorylation in colon cancer stem cells, and inhibited the growth of colon cancer stem cell xenografts in nude mice. Hence, similarly to curcumin, the curcumin analog GO-Y030 has multiple protein targets [36]. In the present study, GO-Y031 targeted both β-catenin and STAT3, which are transcriptional regulators that control various downstream molecules, such as c-Myc, cyclin D1, vascular endothelial growth factor, and survivine, and contribute to cancer cell growth and survival [37, 38]. Furthermore, cross-linking between β-catenin and STAT3 may compensate for single inhibition of one or the other, leading to drug resistance. Thus, simultaneous inhibition of these two major molecules with GO-Y031 may result in improved inhibition of gastric carcinogenesis. Curcumin analogs may also inhibit gastric tumors under conditions of low pH. However, it remains unknown whether curcumin analogs can improve survival in mice with gastric cancer, and GO-Y031 was not curative in Gan mice. Potentially, this may reflect the carcinogenic drive of the transgenes *Ptgs2* (encoding COX2), *Ptges* (encoding microsomal prostaglandin E synthase 1), and *Wnt1* in whole gastric epithelia of Gan mice, which leads to 100 % tumor incidence and more aggressive cancers than the sporadic gastric cancers observed in humans [12]. Nonetheless, the present data warrant assessment of the survival benefits of curcumin analogs in humans, particularly because no inhibitors of β-catenin or STAT3 have been approved for clinical use as chemotherapeutic agents for cancer. Recently, an inhibitor of the CREB-binding protein–β-catenin interaction (ICG-001) was examined in a phase 1/phase 2 study as a potent inhibitor of β-catenin for the treatment of acute lymphoblastic leukemia, chronic myeloid leukemia, pancreatic cancer, and other solid tumors [39, 40]. Numerous studies have shown that STAT3 inhibitors lead to human tumor regression in animal models [22, 41]. However, although preclinical and phase 0 studies of STAT3 inhibitors have progressed with urgency, no such agents have been approved [42]. The present data indicate that the curcumin analog GO-Y031 may be a promising lead agent for the treatment of gastric cancer, acting as a β-catenin and/or STAT3 inhibitor. Although the effects of CD44 inhibition in cancer therapy remain controversial, numerous reports have shown effective reduction of malignancies, including colon, mammary, and ovarian cancers, following treatment with CD44 inhibitors [40]. The downregulation of CD44 after treatment with GO-Y031 suggests that curcumin analogs inhibit gastric cancer stem cells and reflect the inhibition of STAT3 and/or CD44 in colorectal cancer stem cells [22]. In agreement, knockdown of *Stat3* resulted in the downregulation of the stem cell markers Oct-4, Sox-2, and CD44 [43].

Infection with *H. pylori* is a common cause of gastric cancer, and curcumin has been reported to exhibit antimicrobial activity against *H. pylori* [44]. Recently, the antimicrobial activity of curcumin against *H. pylori* was shown to differ between strains, with minimum inhibitory concentrations of 5–50 μg/ml [45]. Moreover, curcumin has been shown to ameliorate *H. pylori* infection in rodents [46]. However, the antimicrobial activity of GO-Y031 against *H. pylori* requires further study.

The safety of diarylpentanoid-type curcumin analogs has been shown in a recent study, and the diarylpentanoid curcumin analog 1,5-bis(4-hydroxy-3-methoxyphenyl)-1,4-pentadiene-3-one (GO-Y022) has been detected in cooked curry [47]. Thus, some deketone curcumin analogs such as diarylpentanoids may be safe to eat.

## Electronic supplementary material

Below is the link to the electronic supplementary material.

Fig. S1 Immunohistochemical analysis of β-catenin (**a**), Ki-67 (**b**), STAT3 (**c**), pSTAT3 (**d**), and p53 (**e**) of gastric cancer in Gan mice. The *bars* represent 50 μm (**a**, **b**, **e**), and 100 μm (**c**, **d**)

Fig. S2 Immunohistochemical analysis of β-catenin (**a**), STAT3 (**b**), and pSTAT3 (**c**) in nontransgenic mice (TIFF 1520 kb)

Fig. S3 Expression of p53 in background normal mucosa from Gan mice fed HFD (**a**) or GO-Y031 (**b**). The *bar* represents 100 μm (TIFF 1520 kb) (TIFF 1520 kb)
