# Supplementary figures and images for: Inhibition of β-catenin and STAT3 with a curcumin analog suppresses gastric carcinogenesis in vivo
Source: Gastric Cancer. 2014 Oct 18;18(4):774–83. doi: 10.1007/s10120-014-0434-3 (PMC4572076; doi:10.1007/s10120-014-0434-3)

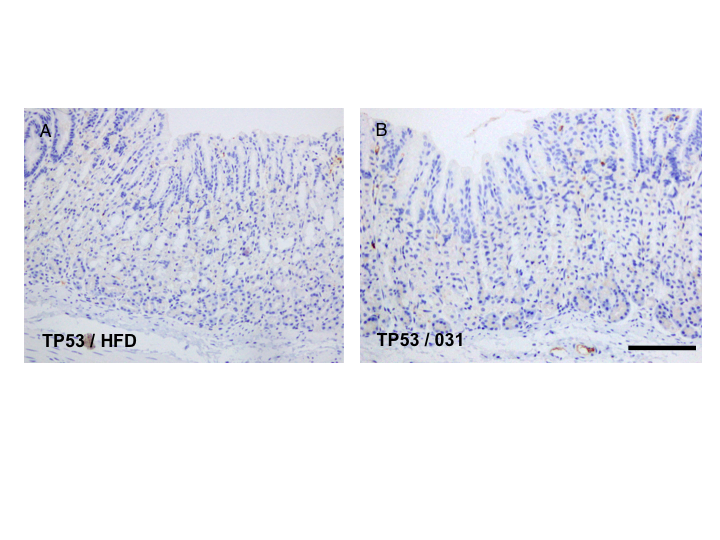

Supplement: Supplementary file 1 — Fig. S1 Immunohistochemical analysis of β-catenin (a), Ki-67 (b), STAT3 (c), pSTAT3 (d), and p53 (e) of gastric cancer in Gan mice. The bars represent 50 μm (a, b, e), and 100 μm (c, d) [file 10120_2014_434_MOESM1_ESM.tif]

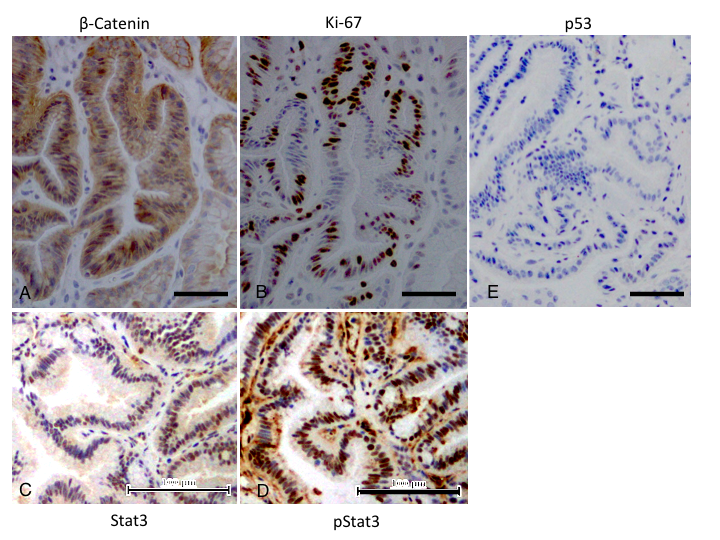

Supplement: Supplementary file 2 — Fig. S2 Immunohistochemical analysis of β-catenin (a), STAT3 (b), and pSTAT3 (c) in nontransgenic mice (TIFF 1520 kb) [file 10120_2014_434_MOESM2_ESM.tif]

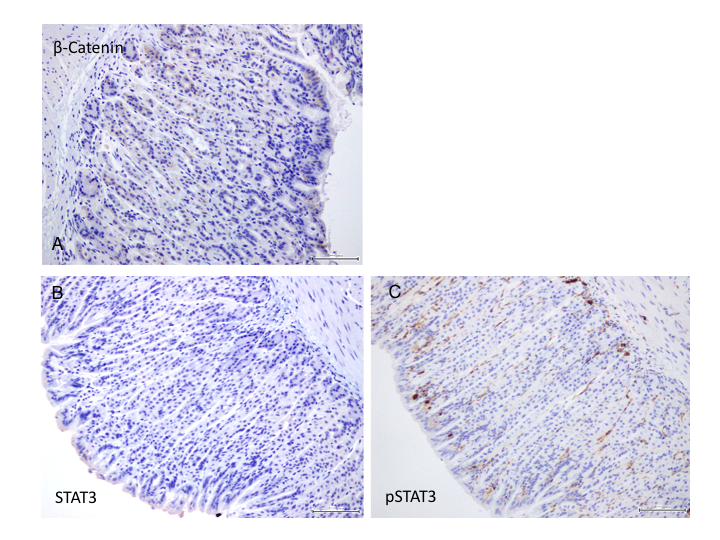

Supplement: Supplementary file 3 — Fig. S3 Expression of p53 in background normal mucosa from Gan mice fed HFD (a) or GO-Y031 (b). The bar represents 100 μm (TIFF 1520 kb) (TIFF 1520 kb) [file 10120_2014_434_MOESM3_ESM.tif]
